# Upper limb paresis due to herpes zoster successfully managed in an outpatient setting: a case report

**DOI:** 10.1186/s13256-026-05978-0

**Published:** 2026-03-31

**Authors:** Yutaka Kozu, Hitoshi Kozu, Yasuhiro Gon

**Affiliations:** 1 Division of Respiratory Medicine, Department of Internal Medicine, Nihon University School of Medicine, 30-1 Oyaguchi Kamicho, Itabashi-Ku, Tokyo 173-8610 Japan; 2 Kozu Medical Clinic, 5-4-7, Wakabayashi, Setagaya-Ku, Tokyo 154-0023 Japan

**Keywords:** Herpes zoster, Brachial plexopathy, Segmental zoster paresis, Upper limb paresis, Cervical spondylosis

## Abstract

**Background:**

Reactivation of the varicella zoster virus can result in a neurological complication, including segmental zoster paresis. Outpatient management of upper limb paresis due to herpes zoster is rarely reported, making this case clinically relevant.

**Case presentation:**

We describe a 70-year-old Japanese woman initially diagnosed with cervical spondylosis who later developed a vesicular rash and progressive left arm weakness. Neurological examination and nerve conduction studies revealed motor involvement consistent with segmental zoster paresis. She was treated with antiviral therapy, physical rehabilitation, analgesics, and adjunctive vitamin supplementation, leading to full recovery.

**Conclusion:**

This case shows the feasibility of outpatient management in select cases of segmental zoster paresis and the importance of timely intervention. Herpes zoster should be considered in the differential diagnosis of acute unilateral arm paresis, especially in cases involving radicular pain.

## Introduction

The varicella zoster virus causes chickenpox during the initial infection and shingles when reactivated, potentially leading to neurological complications [1]. Shingles, or herpes zoster, typically presents as a painful blistering rash confined to a dermatome innervated by a single spinal nerve [2, 3]. This condition is often preceded by pain or tingling and can result in complications such as postherpetic neuralgia, in which pain persists even after the rash has resolved [4]. Although the classic presentation is limited to a single dermatome, individuals with compromised immune systems may experience more widespread clinical manifestations [5–9].

Segmental zoster paresis (SZP) is a rare neurological complication of herpes zoster and is reported in approximately 0.5–5% of cases. It is characterized by motor weakness or paresis in the myotome, corresponding to the dermatomal distribution of the rash [10, 11]. According to published case reports, most patients require inpatient care because of the prolonged disease course and residual weakness [12–20]. Outpatient management appears to be rare.

Herein, we describe the clinical course of an older Japanese woman with SZP of her left arm who was managed entirely in an outpatient setting and made a full recovery. We discuss potential factors that may have contributed to her favorable outcome and highlight the need to consider herpes zoster in the differential diagnosis of acute unilateral arm paresis in older adults.

## Case report

**Fig. 1:**
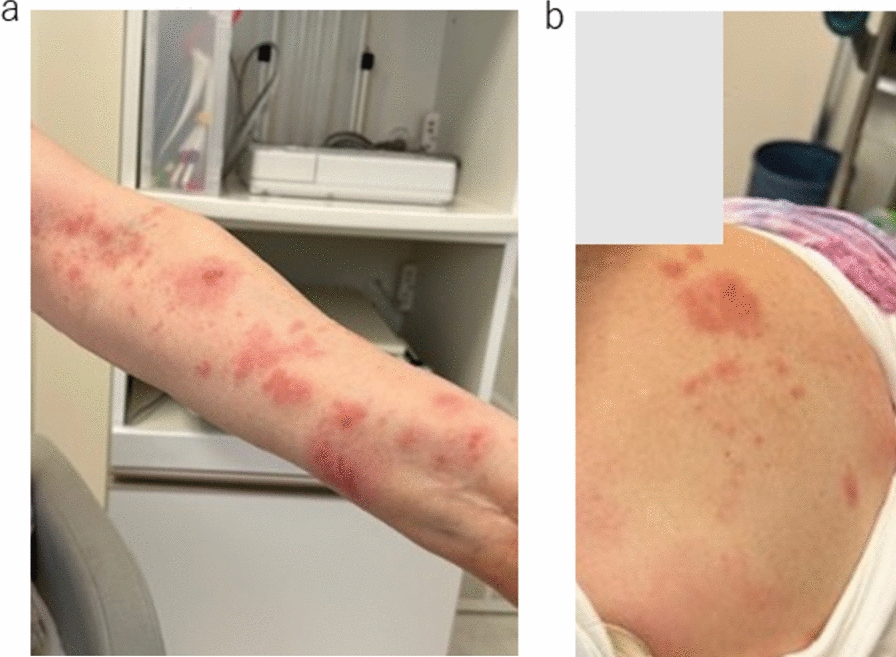
**a**, **b** Herpes zoster vesicular rash of the left arm at the initial consultation

**Fig. 2:**
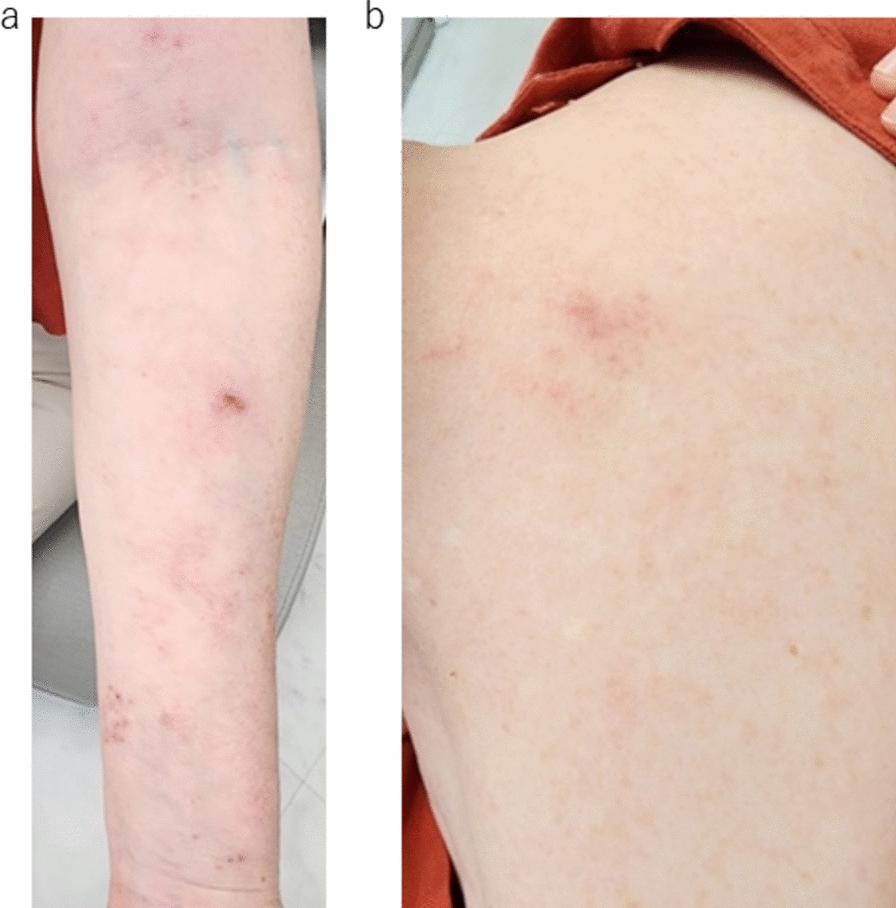
**a**, **b** Photograph taken 2 weeks after the onset of the left arm paresis, showing healing of the rash

A previously healthy 70-year-old Japanese woman presented with pain radiating from the neck to the left arm. She was initially diagnosed with cervical spondylosis and treated conservatively at an orthopedic clinic. However, 2 weeks later, she developed clusters of vesicular eruptions extending from the neck to the left arm, which led to the diagnosis of herpes zoster (Fig. 1). She was treated with valacyclovir (1000 mg three times daily for 7 days) and acetaminophen (500 mg every 6 hours, as needed for pain). No antiviral therapy beyond this guideline-recommended duration was provided. Despite this initial treatment, the symptoms persisted. Therefore, tramadol (50 mg twice daily) and gabapentin (300 mg three times daily) were added to manage the neuropathic pain. In addition, vitamin B12 (1000 µg daily) and vitamin B6 (50 mg daily) were prescribed to support nerve health and limit potential nerve damage. Subsequently, 2 weeks after onset of the rash, the patient experienced a reduction in muscle strength in her left arm (Fig. 2).

Neurological examination revealed reduced strength in the left shoulder flexors and elbow flexors and extensors, predominantly in muscles innervated by the C5–C7 roots (Table 1). Sensation in the corresponding dermatomes was mildly decreased. Nerve conduction studies of both arms revealed decreased compound muscle action potential amplitudes in the left musculocutaneous and axillary nerves, with relatively preserved sensory nerve action potentials, indicating predominant motor involvement of the C5–C7 segments. These clinical and electrophysiological findings, together with the temporal sequence of radicular pain, dermatomal rash, and segmental weakness, led to the clinical diagnosis of SZP. Cervical spine magnetic resonance imaging was performed and showed no evidence of spinal cord compression, myelopathy, or alternative structural causes of the left arm weakness. Because the patient was hemodynamically stable; had no systemic symptoms, cranial nerve involvement, or signs of infection or encephalitis; and had strong family support with easy access to our clinic, she was managed on an outpatient basis. She was followed at regular intervals with repeated neurological examinations to monitor changes in muscle strength and the emergence of new deficits. The patient and her family were instructed to seek urgent medical attention in case of rapid worsening of weakness, new neurological symptoms, or difficulties in performing daily activities. The patient experienced noticeable improvement in muscle strength approximately 6 weeks after the onset of paresis and gradually regained full motor function of the arm. The clinical timeline is presented in Table 2.

**Table 1:** Manual muscle test results (upper limbs)

No.	Motion	Prime mover	Innervating nerve	Spinal segment (affected area)	Test position (grade)	Right	Left
1	Elevation of upper scapula	Trapezius (upper fibers)	Accessory nerve	C5, C6, C7, C8, T1	Rest position	5	5
2	Depression of scapula	Latissimus dorsi	Thoracodorsal nerve	C6, C7, C8	Rest position	5	5
3	Flexion of shoulder joint	Deltoid (anterior fibers)	Axillary nerve	C5, C6	Rest position	2+	5
4	Extension of shoulder joint	Latissimus dorsi	Thoracodorsal nerve	C6, C7, C8	Rest position	5	5
5	Abduction of shoulder joint	Deltoid (middle fibers)	Axillary nerve	C5, C6	Rest position	5	5
6	Adduction of shoulder joint	Latissimus dorsi	Thoracodorsal nerve	C6, C7, C8	Rest position	5	5
7	Horizontal flexion of shoulder	Pectoralis major	Medial pectoral nerve	C5, C6, C7	Rest position	5	5
8	Horizontal extension of shoulder	Deltoid (posterior fibers)	Axillary nerve	C5, C6	Rest position	5	5
9	External rotation of shoulder	Infraspinatus	Suprascapular nerve	C5, C6	Rest position	5	5
10	Internal rotation of shoulder	Subscapularis	Subscapular nerve	C5, C6	Rest position	5	5
11	Flexion of elbow joint	Biceps brachii	Musculocutaneous nerve	C5, C6	Rest position	4+	5
12	Extension of elbow joint	Triceps brachii	Radial nerve	C6, C7, C8	Rest position	5	5
13	Supination of forearm	Biceps brachii	Musculocutaneous nerve	C5, C6	Rest position	5	5
14	Pronation of forearm	Pronator teres	Median nerve	C6, C7	Rest position	5	5
15	Flexion of wrist joint	Flexor carpi radialis	Median nerve	C6, C7	Rest position	5	5
16	Extension of wrist joint	Extensor carpi radialis	Radial nerve	C6, C7, C8	Rest position	5	5
17	Flexion of fingers (proximal interphalangeal [PIP])	Flexor digitorum superficialis	Median nerve	C7, C8, T1	Rest position	5	5
18	Extension of fingers	Extensor digitorum	Radial nerve	C6, C7, C8	Rest position	5	5
19	Flexion of thumb	Flexor pollicis longus	Median nerve	C6, C7, C8	Rest position	5	5
20	Extension of thumb	Extensor pollicis longus	Radial nerve	C6, C7, C8	Rest position	5	5

**Table 2:** Timeline of clinical events

Time point	Clinical event
T0	Initial visit for neck and left upper limb pain; diagnosed with cervical spondylosis
T0 + 2 weeks	Rash appeared; diagnosed with herpes zoster; valacyclovir initiated
T0 + 4 weeks	Paresis developed; nerve conduction studies performed; diagnosed with segmental zoster paresis
T0 + 6 weeks	Muscle strength improved
T0 + several months	Functional recovery

## Discussion

This case highlights the importance of including SZP in the differential diagnosis of acute unilateral limb paresis. In older adults presenting with unexplained upper limb pain and neurological symptoms, maintaining a high index of suspicion for herpes zoster is crucial, even if initial imaging suggests cervical spondylosis. Early identification of subtle dermatological signs will help avoid misdiagnosis and treatment delays. This patient’s advanced age made her more susceptible to herpes zoster [21, 22], and the involvement of multiple nerve segments (C5, C6, and C7) likely contributed to the severity of neurological deficit [23].

Several differential diagnoses were considered. Cervical radiculopathy due to degenerative spine disease was initially suspected; however, the subsequent appearance of a dermatomal vesicular rash and the distribution of weakness made this diagnosis unlikely. In addition, cervical spine magnetic resonance imaging was performed and showed no evidence of spinal cord compression, myelopathy, or other structural abnormalities that could explain the motor weakness. Brachial neuritis (Parsonage–Turner syndrome) was also considered, but the close temporal association between herpes zoster reactivation and the onset of weakness, together with the electrophysiological pattern, made SZP more likely. Central nervous system disorders such as stroke were deemed unlikely because no supraspinal signs, facial weakness, or speech disturbances were present.

To better understand the clinical course and outcomes, we conducted a literature review of previously reported cases of SZP, of which nine [12–20] were available for detailed analysis on the basis of accessibility and completeness of clinical data. A summary of these cases is provided in Table 3. Among the nine cases reviewed, three involved the right arm, five involved the left arm, and one was bilateral. Six of the nine patients (67%) were female individuals. Unlike the treatment of the present case, none of the reviewed case reports mentioned supplementation with vitamins B6 and B12 as adjunctive therapy to mitigate nerve inflammation. Most patients required inpatient care. In contrast, the patient in the present case was managed as an outpatient, demonstrating the feasibility of outpatient management in select cases when early diagnosis and timely intervention are achieved.

**Table 3:** Summary of reported segmental zoster paresis cases

Title	Age (years)/sex	Main symptoms	Paresis level	Side	Duration	Treatment	Outcome	References	Setting
Upper limb segmental zoster paresis (SZP)	70/female	Pain and weakness in right shoulder	C5–7	Right	4 months	Analgesics, tricyclic antidepressant, physical therapy	Residual weakness and pain	Özlem *et al*. [12]	Inpatient
SZP with diaphragmatic paresis	73/female	Pain and weakness in left shoulder	C5–6	Left	1 year	Not specified	Full recovery	Bahadir *et al*. [13]	Inpatient
Pediatric SZP	10/male	Rash, pain	C5, T5 (right), C2–T1 (left)	Left	6 months	Acyclovir, morphine, physical therapy	Strength ≥ 4/5	Ruppert *et al*. [14]	Inpatient
SZP in cervical spinal stenosis	72/female	Pain in left shoulder	C4–6	Left	6 months	Acyclovir, antidepressants, nerve block, pregabalin, physical therapy	Strength 3/5	Kang *et al*. [15]	Not specified
Right shoulder SZP	88/male	Pain in right shoulder	C5–6	Right	Not specified	Acyclovir, methylprednisolone	Gradual improvement	Namekawa *et al*. [16]	Inpatient
MRI-confirmed SZP	79/female	Pain in left shoulder	C5–T2	Left	17 months	Acyclovir, analgesics, nerve block, prednisolone	Improvement	Jihwan *et al*. [17]	Inpatient
Cervical root involvement in SZP	72/female	Pain in left shoulder	C5–6	Left	7 months	Acyclovir, methylprednisolone, physical therapy	Full recovery	Wada *et al*. [18]	Inpatient
Temporary spinal stimulation for SZP	62/female	Pain in right upper limb	C4	Right	3 months	Acyclovir, analgesics, spinal cord stimulation, physical therapy	Persistent pain	Yamaguchi *et al*. [19]	Inpatient
Left upper limb pain in SZP	72/male	Pain in left upper limb	C4	Left	2 months	Acyclovir, prednisolone, analgesics, physical therapy	Residual pain and weakness	Chen *et al*. [20]	Inpatient

Early intervention with antiviral agents and neuroprotective treatments, including analgesics and nonsteroidal anti-inflammatory drugs, forms the cornerstone of herpes zoster management. Comprehensive pain management, including the use of carbamazepine or gabapentinoids, is essential to address nerve irritation caused by inflammation.

At our clinic, we routinely administer vitamins B6 and B12 as adjunctive therapy because of the potential neurotrophic and anti-inflammatory effects suggested by experimental studies [24–29], although limited clinical data are available. In this patient, these vitamins were given as adjunctive therapy alongside antiviral agents, analgesics, and physical therapy. However, their specific contribution to the favorable outcome in this case cannot be determined. None of the 47 patients treated for herpes zoster at our clinic between December 2020 and December 2024 developed postherpetic neuralgia. However, owing to the small cohort size and the lack of a control group, these observations should be interpreted with caution and cannot be taken as evidence of efficacy. Importantly, the use of vitamin B6 and B12 supplementation in this case should not be interpreted as a treatment recommendation but rather as an individualized supportive measure. Early antiviral therapy and comprehensive pain management remain the key evidence-based strategies for managing herpes zoster.

## Conclusion

This case highlights the importance of careful diagnosis and individualized management of SZP in older adults. Although the patient developed marked upper limb paresis that would usually prompt inpatient care, frequent outpatient visits, timely antiviral therapy, multimodal pain control, vitamin supplementation as adjunctive therapy, and early rehabilitation were associated with gradual but complete recovery. This experience suggests that outpatient management may be feasible in carefully selected, clinically stable patients with SZP; however, inpatient treatment, including intravenous antivirals and steroids, remains essential for more severe or rapidly progressive cases. Further accumulation of case reports and larger studies are needed to establish evidence-based guidelines for the management of SZP.

## Data Availability

Data sharing is not applicable to this article as no datasets were generated or analyzed during the study.

## Abbreviation

SZP: Segmental zoster paresis
